# Ammonium-Containing Methacrylic Polymer Brushes with Adjustable Hydrophilicity: Synthesis and Properties in Aqueous Solutions

**DOI:** 10.3390/polym17091200

**Published:** 2025-04-27

**Authors:** Denis Kamorin, Alexander Simagin, Oleg Kazantsev, Maria Savinova, Maria Simonova, Denis Sadkov, Ildar Arifullin, Yaroslav Dolinov

**Affiliations:** 1 Research Laboratory “New Polymeric Materials”, Nizhny Novgorod State Technical University n.a. R.E. Alekseev, 24 Minin Street, 603950 Nizhny Novgorod, Russia; 2 Laboratory of Biomimetic Polymer Materials, Branch of Petersburg Nuclear Physics Institute Named by B.P. Konstantinov of National Research Centre «Kurchatov Institute»—Institute of Macromolecular Compounds, Bolshoy Prospekt 31, 199004 Saint Petersburg, Russia

**Keywords:** oligo(ethylene glycol) methacrylate, dialkylaminoalkyl methacrylamide, RAFT polymerization, amphiphilic polymers, thermoresponsive polymers, micelles

## Abstract

Reversible addition–fragmentation chain-transfer (RAFT) polymerization was used to synthesize novel thermoresponsive cationic molecular brushes with high yields—namely of copolymers of methoxyoligo(ethylene glycol) methacrylate, alkoxyoligo(ethylene glycol) methacrylate, and N-methacryloylaminopropyl-N,N-dimethyl-N-propylammonium bromide. Controlled polymerization yielded polymers with a molecular weight dispersity of ≤1.3 and conversions exceeding 80%. The influence of the cationic molecular brushes’ composition on their solubility in water and organic solvents, interfacial tension at the water–oil interface, and aggregation behavior in aqueous solutions was investigated. For the first time, we report the design of thermoresponsive cationic molecular brushes combining an antibacterial potential and tunable hydrophilic–hydrophobic properties, enabling highly precise control over their LCST behavior (17–68 °C) through composition tuning. The solubilization capacity of the hydrophobic compounds of brush micelles in water increased with the hydrophobic comonomer content. These polymers exhibited interfacial activity, significantly reducing the water–oil interfacial tension, with critical micelle concentrations (CMCs) of 3–10 mg/L. It was shown that the amphiphilic properties of the copolymers in aqueous solutions can be easily tuned in a desired direction by varying the content of the comonomer units. The obtained data indicate the potential of the polymers as micellar nanocarriers for controlled drug delivery.

## 1. Introduction

One of the approaches to improving the bioavailability of poorly water-soluble hydrophobic drugs, as well as to achieving targeted and controlled drug release in the body, is the use of polymer-based micellar delivery systems [1,2,3]. The requirements for micellar nanocarriers include biocompatibility, a high drug-loading capacity, surface activity, low critical micelle concentration (CMC) values, and high micelle stability [1,2,3,4]. Additionally, micelles gain further advantages when the polymers exhibit stimuli-responsive properties [1,5] and intrinsic biological activity [6,7]. Achieving all these characteristics can be accomplished by selecting the optimal composition of the amphiphilic polymer.

A large number of reviews and exploratory studies have been devoted to the synthesis and properties of polymers with the molecular brush architecture [8,9,10,11,12,13,14]. Molecular brushes consist of a linear backbone with densely grafted polymeric side chains [8,15,16]. Due to steric repulsion between the densely grafted side chains, molecular brushes adopt a cylindrical shape in solutions [17,18,19]. Unlike polymeric micelles formed by amphiphilic block copolymers, molecular brushes with block-like side chains form stable, unimolecular, cylindrical micelles [20,21,22], which are capable of maintaining their structure even under extreme dilution, which is important for their use in drug delivery. Moreover, cylindrical molecular brushes have an increased surface area and volume compared to spherical particles, making them more attractive for surface adsorption and drug encapsulation [18]. The presence of polymer fragments with stimuli-responsive properties in molecular brushes imparts the ability to undergo conformational transitions in response to changes in the external environment (temperature, pH, salt concentration, electromagnetic fields, etc.) [15,17].

Due to these properties, molecular brushes have garnered significant practical interest, particularly in various fields of medicine, including drug delivery, gene delivery, and peptide delivery [10,23]. Additionally, amphiphilic polymer brushes can serve as polymeric surfactants [24,25] and be used in the creation of elastomers [14,26,27], coatings [28,29], polymeric catalytic systems [30,31], and other industrial applications [13,14].

Advances in the synthesis of molecular brushes by the so-called “grafting-through” method have been described in a review [9] that uses the example of polymers with polyethylene glycol side chains. Such polymers are widely used in the development of polymeric nanocarriers for drug delivery [32,33], which can circulate in the bloodstream for extended periods of time without undesirable interactions with blood components, providing the polymers with good biocompatibility and biodegradability [32,33,34].

Amphiphilic (co)polymers of methoxyoligo(ethylene glycol) methacrylates (MOEGMs) have been intensively studied over the past 20 years, and many reviews have been published on this topic (e.g., [9]). The thermoresponsive properties of MOEGM copolymers can further enhance the efficacy of these polymers in drug delivery within the body, which is why many researchers prefer such macromonomers [35]. Their lower critical solution temperature (LCST) values can be easily adjusted by varying the number of oxyethylene fragments in the initial MOEGM (which can reach 50 or more) and by incorporating hydrophobic comonomer units [36,37].

MOEGM copolymers with higher alkyl methacrylates or alkoxyoligo(ethylene glycol) methacrylates (AOEGMs), with a random distribution of hydrophobic units along the macromolecular chain, are capable of forming micellar structures in aqueous solutions with a hydrophobic core containing alkyl fragments and a hydrophilic polyethylene glycol shell [38,39,40,41,42]. Such copolymers exhibit very low CMC values and can effectively solubilize hydrophobic compounds in aqueous environments. Similar properties are exhibited by copolymers of MOEGM and higher AOEGM (AOEGM, where n-alkyl groups C_12_ and above are separated from the main macromolecular chain by an oligoethylene glycol fragment) [41]. For the synthesis of copolymers based on MOEGMs or AOEGMs, various methods of radical polymerization, including controlled polymerization, can be employed [40,42,43]. Reversible addition–fragmentation chain transfer (RAFT) polymerization allows for the synthesis of statistical or block copolymers with a predetermined molecular weight and narrow molecular weight distribution [44,45].

The successful use of molecular brushes containing cationic units as carriers for drug delivery and gene delivery into cells has been reported [46,47,48,49]. The introduction of cationic ammonium units into amphiphilic, stimuli-responsive polymers can enhance the retention of certain drugs by the polymeric delivery system. For example, it was found that the electrostatic interactions between cationic polymers and DNA are significantly stronger than those between non-cationic polymers and DNA, due to the presence of permanent positive charges in cationic polymers [50]. Another important aspect is the strong antibacterial properties exhibited by ammonium-containing polymer brushes [51,52]. Due to their high antimicrobial activity [53], cationic molecular brushes based on 3-dimethylaminoethyl methacrylate (DMAEM) have been proposed for the development of environmentally friendly nanoantibiotics [51].

In addition to (meth)acrylic, amino-containing esters such as DMAEM, another widely used monomer for obtaining cationic, stimuli-responsive polymers is the amino-containing N-[3-(dimethylamino)propyl]methacrylamide (DMAPMA), which is active in radical copolymerization with methacrylic esters [54]. Unlike DMAEM, DMAPMA has sufficient hydrolytic stability [55], is produced industrially, and can easily be converted into a quaternary ammonium compound by alkylation of the tertiary amine with alkyl halides. The cationic monomer N-methacryloylaminopropyl-N,N-dimethyl-N-propylammonium bromide (DMq) can be synthesized [56] from DMAPMA, which is widely used for obtaining thermo- and pH-sensitive polymers, including those for drug and gene delivery [57,58].

**Figure 1:**
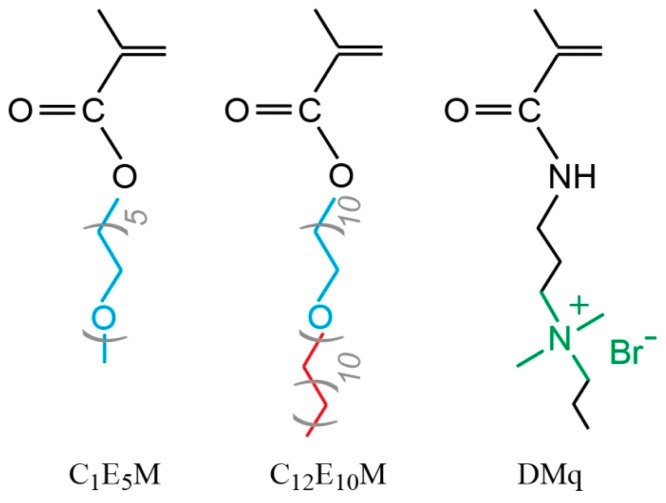
Chemical structures of monomers.

Despite significant advances in polymer-based drug delivery systems, the development of precisely tunable micellar carriers combining three critical functionalities remains challenging: (1) temperature-responsive solubility, (2) controlled drug loading/release via balanced hydrophobicity, and (3) inherent antimicrobial activity. This work addresses these needs through the design of novel cationic molecular brushes via the RAFT polymerization of three functional monomers (Figure 1): DMq (providing antimicrobial quaternary ammonium groups), C_1_E_5_M (MOEGM with five ethylene glycol units, offering thermoresponsive properties), and C_12_E_10_M (AOEGM with ten ethylene glycol units and a C_12_ alkyl fragment, ensuring hydrophobic micelle core formation).

Notable features of this study include a one-pot synthesis strategy enabling the efficient preparation of amphiphilic thermoresponsive bottlebrush terpolymers via RAFT polymerization, with precise control over monomer composition, molecular weight, and brush architecture. A comprehensive study of macromolecular behavior and aggregates in aqueous solutions reveals systematic correlations between polymer composition and aggregation properties, including CMC, LCST, and interfacial activity. The established structure–property relationships provide a blueprint for developing next-generation therapeutic carriers with programmable behavior in physiological conditions.

## 2. Materials and Methods

### 2.1. Materials

In this study, a commercial macromonomer MOEGM with an average number of ethylene glycol units equal to five, C_1_E_5_M (average M_n_ 300, Sigma Aldrich, Burlington, MA, USA), was used. DMq and C_12_E_10_M were obtained according to the methods described in the Supporting Information. The cationic methacrylic monomer DMq was synthesized in a stirred reactor by the alkylation of DMAPMA (99.0 wt.%, Sigma Aldrich) with propyl bromide (reagent grade). C_12_E_10_M was obtained by the esterification of methacrylic acid with the corresponding alcohol. Prior to use, polymerization inhibitors were removed from the macromonomers by passing it through activated aluminum oxide. The RAFT agent 4-cyano-4-(dodecylsulfanylthiocarbonyl)sulfanylpentanoic acid was synthesized according to the method described in [59].

### 2.2. Synthesis of Polymers

The homopolymer of C_1_E_5_M and the copolymer of C_1_E_5_M and DMq were synthesized in a jacketed reactor (Figure S1, Supporting Information) equipped with a reflux condenser, magnetic stirrer, and nitrogen inlet, using dimethylformamide (DMF) as a solvent at 70 °C (total monomer concentration: 30 wt.%). Then, 2,2’-Azobisisobutyronitrile (AIBN) was used as the initiator. RAFT polymerization for the synthesis of terpolymers C_1_E_5_M-C_12_E_10_M-DMq was carried out under the same conditions in the presence of 4-cyano-4-(dodecylsulfanylthiocarbonyl)sulfanylpentanoic acid as the chain transfer agent (CTA).

A typical RAFT polymerization procedure was performed as follows: In a reactor with stirring (~400 rpm), AIBN (0.021 g, 0.126 mmol), CTA (0.202 g, 0.5 mmol), C_1_E_5_M (3.380 g, 11.3 mmol), C_12_E_10_M (0.446 g, 0.64 mmol), and DMq (0.184 g, 0.63 mmol) were dissolved in DMF (9.4 g). The reaction mixture was purged with N_2_ for 15 min, and polymerization was initiated by circulating hot water through the reactor jacket. Throughout the polymerization, the reaction mixture was maintained under a nitrogen atmosphere.

After synthesis, reaction mixtures were dialyzed (MWCO 2kDa, Orange Scientific, Brussels, Belgium) against either water (for the C_1_E_5_M homopolymer and C_1_E_5_M-DMq copolymer) or ethanol (for terpolymers containing C_12_E_10_M units), followed by polymer precipitation from toluene solution into cold hexane.

During polymerization, the current concentrations of methacrylic esters in the reaction mixtures were determined using gas and liquid chromatography (see Supporting Information), and data on monomer consumption were used to calculate conversions. Then, ^1^H NMR spectra were recorded at 25 °C in deuterated chloroform on an Agilent DD2 400 spectrometer at a resonance frequency of 400 MHz. The molecular weight characteristics of the polymers were determined by size-exclusion chromatography (SEC; see Supporting Information for details).

### 2.3. Methods for Investigating Polymer Properties in Solutions

To evaluate the solubility of the polymers at 25 °C in water and organic solvents, a polymer concentration of 1 wt.% was used [60].

The interfacial tension of the polymers at the water–toluene interface was determined using the stalagmometric method [61] at 25 °C and various polymer concentrations. Measurements were performed using a stainless steel capillary with a diameter of 0.7 mm.

The cloud point temperature (T_cp_) of the polymers at various concentrations in water and saline solutions (0.1 M NaCl) was determined by turbidimetry. The T_cp_ was taken as the maximum of the first derivative of the S-shaped dependence of light transmittance on temperature [62]. The turbidimetric determination of the T_cp_ was performed on a KFK-2MP photocolorimeter at 540 nm and a heating rate of 0.3 °C/min, which is close to the recommended conditions [63].

The critical micelle concentration (CMC) of the polymers in water was determined using the pyrene probe method by monitoring the change in the ratio of pyrene emission band intensities as a function of polymer concentration using an RF-6000 spectrofluorometer (Shimadzu, Japan) [64]. The transition of pyrene molecules from a highly polar aqueous environment to a less-polar organic phase leads to a change in the ratio of emission intensities, I_1_ and I_3_, of pyrene at two emission bands (at λ_1_ = 372 nm and λ_3_ = 382 nm). As a result, when the CMC is reached, a break occurs in the plot of I_1_/I_3_ versus the polymer concentration, caused by the initial incorporation of pyrene molecules into the forming micelles, which leads to a decrease in the I_1_/I_3_ value. CMC was determined as a concentration corresponding to the inflection point at which I_1_/I_3_ started to decrease. Aqueous solutions of polymers with 10 different concentrations in the range of 1 × 10^−6^ ÷ 0.5 mg/mL were prepared by dissolving the polymers in aqueous pyrene solutions (6 × 10^−7^ M).

To evaluate the capacity of micelles for a hydrophobic marker (pyrene), a weighed amount of pyrene was added to an aqueous polymer solution (0.1 wt.%). The solution was then treated in an ultrasonic bath (Ultrasound Cleaner T-020ST, frequency 40 kHz, power 120 W) to enhance the transfer of pyrene into the hydrophobic cores of the micelles. The remaining undissolved marker was removed by filtration. To determine the content of solubilized pyrene in the solution, a sample was dissolved in acetonitrile, and the absorption intensity at 334 nm was measured using a Shimadzu UV-1800 spectrophotometer.

Light scattering measurements were performed using a Photocor Complex instrument (Photocor Instruments Inc., Moscow, Russia) equipped with a Photocor-DL diode laser (λ = 659.1 nm) and a Photocor-PC2 correlator (288 channels). The data were processed using DynaLS software (v. 8.2.3, SoftScientific, Tirat Carmel, Israel). The system was calibrated using benzene as a standard (Rayleigh ratio R_V_ = 2.32∙10^−5^ cm^−1^).

The absolute molar masses and hydrodynamic characteristics (*R*_h−D_) of the samples were determined in acetonitrile (density ρ_0_ = 0.78 g∙cm^−3^, dynamic viscosity η_0_ = 0.34 cP, and refractive index n_0_ = 1.341) and water (_0_ = 1.00 gcm^−3^, η_0_ = 0.98 cP, and n_0_ = 1.333) using static (SLS) and dynamic (DLS) light-scattering methods. All the measurements were performed at 21 °C. Solutions were filtered through Millex PTFE filters (0.45 μm pore size) for organic solvents and Chromafil Xtra PA-20/25 filters (0.45 μm pore size) for aqueous solutions.

For the investigated acetonitrile solutions, the distribution of the light-scattering intensity (I) versus the hydrodynamic radius (*R*_h−D_) was unimodal. The *R*_h−D_ values were determined across a wide concentration range and extrapolated to zero concentration to obtain the hydrodynamic radius of individual macromolecules (*R*_h−D_). As established, the translational diffusion coefficient (D_0_) and friction coefficient (f) of macromolecules are related to the *R*_h−D_ through the Stokes–Einstein equation:

(1) $$D_{0}=\frac{k_{B}T}{f}=\frac{k_{B}T}{6\pi \eta_{0}R_{h-D}}$$

where k_B_ is the Boltzmann constant, and *T* is the absolute temperature. SLS measurements were performed at a 90° angle since no angular dependence of the scattered light intensity was observed. The results were analyzed using the Debye method, with the weight-average molar mass, *M*_w_, and second virial coefficient, *A*_2_, calculated according to the following equation:

(2) $$\frac{cH}{I_{90}}=\frac{1}{M_{w}}+2A_{2}c$$

where

H is the optical constant.

*I*_90_ is the excess intensity of light scattered at 90°.

No angular dependence of the light-scattering intensity was observed. The weight-average molar masses, M_w_, and second virial coefficients, *A*_2_, were determined using the Debye method.

Refractive index increments (*dn/dc*) were measured using an RA-620 refractometer (KEM). The dn/dc values were obtained from the slope of concentration-dependent plots of Δn = *n* − *n_0_,* where *n* and *n*_0_ represent the refractive indices of the solution and solvent, respectively.

Intrinsic viscosity ([η]) measurements were conducted in chloroform at 21 °C using an Ostwald-type Cannon–Manning capillary viscometer (Cannon Instrument Company, State College, PA, USA).

The concentration dependence of the reduced viscosity (η_sp_/c) was analyzed using the Huggins equation:

(3) $$\frac{\eta_{sp}}{c}=\left[\eta \right]+k_{H}{\left[\eta \right]}^{2}c$$

where

[η] is the intrinsic viscosity.

k_H_ is the Huggins constant.

Aqueous solutions of C_1_E_5_M-C_12_E_10_M-DMq samples were investigated using static (SLS) and dynamic (DLS) light-scattering techniques, along with turbidimetry, employing the aforementioned Photocor Complex instrument. The system was equipped with a Photocor-PD detector for measuring the transmitted light intensity.

The solution temperature (*T*) was varied discretely in 1.0–5.0 °C increments, with a temperature control precision of ±0.1 °C over a range of 15–79 °C. Upon reaching each target temperature, all the measured parameters evolved over time until reaching steady-state values (characteristic time *τ*_eq_). Under equilibrium conditions (time-independent solution parameters), the following quantities were determined: scattered light intensity, *I*; optical transmittance, I*; hydrodynamic radii, *R*_h-f_ (fast mode) and *R*_h-s_ (slow mode), of scattering species; and their relative contributions, *S*_i_, to the total scattering intensity. The S_i_ values were calculated from the area under the corresponding *R*_h_ distribution peaks. Measurements were performed at scattering angles between 45° and 135° to verify the diffusive nature of the observed modes. To maintain the detector linearity, the scattered light intensity was attenuated using optical filters and laser power reduction, keeping the photon count rate below 1.2 MHz.

## 3. Results

### 3.1. Investigation of Polymerization

As a result of studying the solution radical copolymerization of the macromonomers C_1_E_5_M and C_12_E_10_M with the cationic monomer DMq, the synthesis conditions ensuring a controlled process were determined, and polymers with varying monomer compositions were obtained.

**Figure 2:**
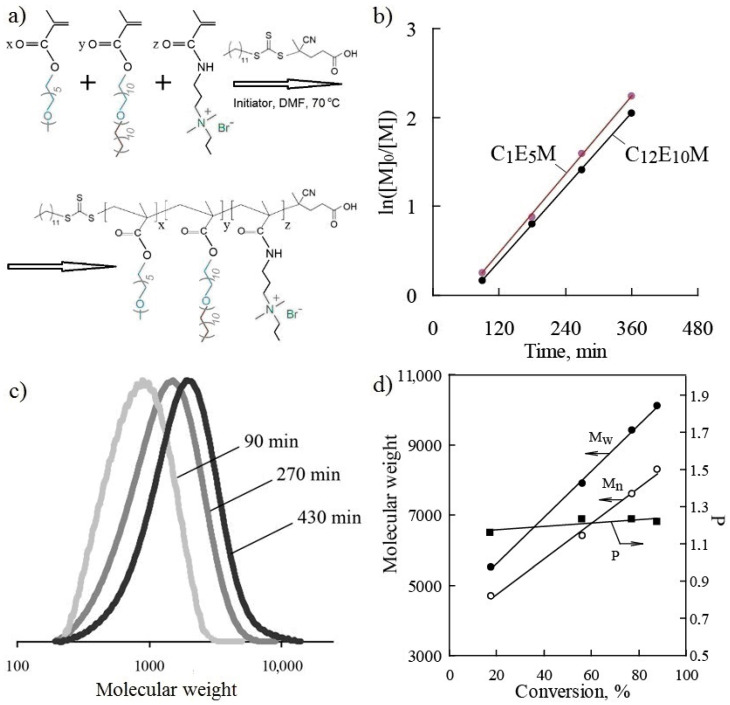
The investigation of the synthesis of terpolymers: the reaction scheme (**a**); a plot of ln([M]_0_/[M]) vs. time (**b**); molecular weight distribution curves as a function of the synthesis time (**c**); the values of weight-average, M_w_, and number-average molecular weights, M_n_, and polydispersity, P, at various degrees of conversion (**d**) for the terpolymer C_1_E_5_M-C_12_E_10_M-DMq (60:35:5).

The terpolymers were synthesized via RAFT polymerization in the presence of the chain transfer agent (CTA) 4-cyano-4-(dodecylsulfanylthiocarbonyl)sulfanylpentanoic acid and AIBN as the initiator. The CTA has previously been shown to be highly effective in the polymerization of both methacrylic esters and amides [65,66,67,68]. The synthesis scheme for the terpolymers is presented in Figure 2a.

The investigation of polymerization kinetics revealed high monomer activity under RAFT polymerization conditions. The dependencies of ln([M]_0_/[M]) on time for C_1_E_5_M and C_12_E_10_M, presented in Figure 2b, indicate a steady-state polymerization regime where termination reactions are balanced by the formation of radicals from the initiator. The controlled polymerization regime, proceeding to high conversions, is confirmed by the linear nature of the kinetic curves in semi-logarithmic coordinates. When extrapolating the dependencies, the lines do not pass through the origin, which occurs in cases of slow initiation and the presence of an inhibition period.

For the macromonomers C_1_E_5_M and C_12_E_10_M, similar polymerization rates were observed, ensuring the absence of significant changes in monomer composition (gradient in the distribution of monomer units) along the macromolecule. The final composition of the studied terpolymers was confirmed by an elemental analysis, ^1^H NMR, and IR spectroscopy (see Supporting Information) and is presented in Table 1.

The selected RAFT polymerization conditions ensured an increase in the molecular weight of the polymers with increasing conversion. Figure 2c shows examples of molecular weight distribution curves of the terpolymer as a function of synthesis time, while Figure 2d illustrates the change in the weight-average and the number-average molecular weights (M_w_ and M_n_), as well as the polydispersity index (P = M_w_/M_n_), as a function of monomer conversion. The controlled polymerization regime was confirmed by the linear increase in the polymer molecular weight with the conversion and the narrow molecular weight distribution, with the polydispersity index not exceeding 1.3 (Table 1). During synthesis, the polydispersity of the molecular weight slightly increased with the conversion (Figure 2d). In all the cases, the final monomer conversions exceeded 85% (Table 1), while maintaining a high degree of molecular weight control.

The theoretical molecular weights (Mth), calculated based on the initial molar ratio of the monomers, CTA, and the initiator ([M]:[CTA]:[I]) and considering the conversion, were found to be close to the experimentally determined values (Table 1). The experimental relative molecular weights were obtained by size-exclusion chromatography using polymer standards of a different chemical nature (polystyrene), which may account for some discrepancies between the experimental and calculated values.

In addition to the studied terpolymers, the homopolymer C_1_E_5_M and the binary copolymer C_1_E_5_M-DMq were synthesized under radical polymerization conditions as reference materials. The synthesis conditions, monomer compositions, conversions, and molecular weight characteristics of the polymers are presented in Table 1.

### 3.2. Investigation of Polymer Properties

The solubility of the polymers was determined in a wide range of organic solvents and water at 25 °C. Table 2 lists the solvents used, ordered by the increasing dielectric constant (ε). The monomer DMq is highly soluble in more-polar solvents with ε values above 10 and in chloroform (ε = 4.8). The homopolymer C_1_E_5_M was insoluble only in the most hydrophobic solvent used—hexane (ε = 1.9). The terpolymers and the copolymer C_1_E_5_M-DMq were insoluble not only in hexane but also in cyclohexane (ε = 2.0), regardless of the content of hydrophobic monomer units within the studied range of ratios. Thus, the introduction of up to 20% cationic DMq units only slightly reduces the range of suitable solvents for MOEGM-based copolymers, while the incorporation of up to 35% hydrophobic n-alkyl groups in the form of C_12_E_10_M units does not lead to the loss of copolymer solubility in polar organic solvents and water. In solvents with an ε value of greater than 10 (isopropanol, acetone, ethanol, dimethylformamide, acetonitrile, and water), all the studied polymers were soluble.

Extensive studies were conducted on the molar masses and hydrodynamic characteristics of the polymers. The *M*_w_ and *R*_h−D_ values are presented in Table 3. Note that the second virial coefficients, *A*_2_, were positive, indicating that acetonitrile is a thermodynamically good solvent for the investigated polymers, and increasing the C_12_E_10_M mole fraction did not alter the *dn/dc*. Table 1 and Table 3 demonstrate good agreement between the experimentally determined and calculated molecular masses.

For all the samples, the values of intrinsic viscosity [η] and the hydrodynamic radii, *R*_h−D_, were relatively small, indicating the compact dimensions and symmetrical shapes of the macromolecules. The low [η] and *R*_h−D_ and high the Huggins constant, K_H_, suggest a high intramolecular density and a low asymmetry of the C_1_E_5_M-C_12_E_10_M-DMq macromolecules in acetonitrile. Additionally, low values of the hydrodynamic invariant, A_0_, refs. [69,70,71,72] were observed for the studied copolymers. A_0_ was calculated using experimental values of M_w_, [η], and D_0_ via Equation (4):

(4) A0=η0D0Mη/10013T

The value of A_0_ remains constant over a wide range of polymer molar masses and depends on the molecular conformation and architecture. Notably, for flexible-chain and rigid-chain polymers, the average experimental values of the hydrodynamic invariant differ by nearly 20%: *A*_0_ = 3.2 × 10^−10^ and 3.8 × 10^−10^ erg × K^−1^mol^−⅓^ [73]. For the studied samples in acetonitrile, *A*_0_ = 2.4 × 10^−10^ erg × K^−1^mol^−⅓^. Low values of *A*_0_ (≤2.8 × 10^−10^), which are below the theoretical prediction for a rigid sphere, are characteristic of polymers with complex architectures, such as molecular brushes, hyperbranched polymers, and star-shaped polymers [74,75,76].

**Figure 3:**
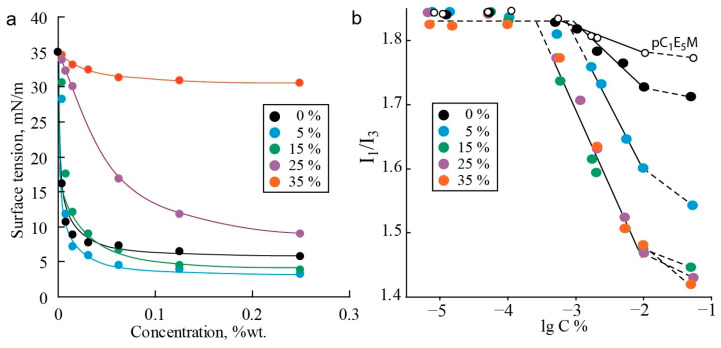
Dependence of interfacial tension on concentration for terpolymers with varying contents of C_12_E_10_M units (mol.%) in a toluene–water biphasic system (**a**). The determination of CMC values from the dependence I_1_/I_3_ of pyrene on the concentration of the polymers (**b**).

An important aspect in the context of micellar drug delivery is the biodistribution of nanocarriers in the human body and their behavior in biphasic water–lipid systems, as well as the permeability of the nanocarrier through biological membranes [2,77]. Therefore, it is of interest to study the distribution of polymers between phases and their interfaces, as well as the surface-active properties of the investigated systems. Amphiphilic polymers exhibit high interfacial activity and significantly reduce the tension at the water–oil interface [78,79]. Figure 3a shows the interfacial tension isotherms for polymers in toluene–water systems. The copolymer C_1_E_5_M-DMq demonstrates significant interfacial activity at the water–oil interface, surpassing that of the original methoxyoligoethylene glycol methacrylates and DMAPMA [80]. This may indicate a high tendency of the obtained polymers to aggregate in aqueous solutions.

It was also found that the content of the hydrophobic macromonomer C_12_E_10_M significantly influences the interfacial tension. The introduction of 5% C_12_E_10_M units endows the copolymer with interfacial activity exceeding that of the C_1_E_5_M-DMq copolymer and comparable to the C_1_E_5_M homopolymer (limiting tension value of 5 mN/m). However, further increasing the content of C_12_E_10_M units from 5% to 35% reduces the interfacial activity of the polymers in the toluene–water system. Apparently, the introduction of a small percentage of hydrophobic monomer units modifies the polymer similarly to other water-soluble acrylic polymers, where the incorporation of a small fraction of monomers with alkyl groups imparts special aggregation and rheological properties to the polymers [81,82]. Further increasing the proportion of the low-polarity component hinders the localization of the polymer at the phase interface, and the surface activity decreases. In experiments with the “toluene—saline aqueous solution (0.1 M NaCl)” system, the differences between the polymers are leveled, and the isotherms of all the polymers reach a plateau in the range of 4–8 mN/m. This indicates that the studied systems can provide the required behavior for drug delivery carriers in biphasic water–lipid systems due to their pronounced surface-active properties.

For the synthesized polymers, the critical micelle concentration (CMC), the capacity of micelles for a low-molecular-weight hydrophobic compound (pyrene, used as a model of a drug), and the hydrodynamic radii (*R*_h_) of the polymer associates in aqueous solutions were determined (Table 3).

The determination of the CMC based on the ratio of fluorescence emission band intensities of the marker is illustrated in Figure 3b. The transition of pyrene molecules from a highly polar aqueous environment to a less-polar organic phase leads to a change in the ratio of emission intensities I_1_ and I_3_ at two emission bands (at λ_1_ = 372 and λ_3_ = 382) [83]. As a result, when the CMC is reached, a break occurs in the plot of I_1_/I_3_ versus the polymer concentration, caused by the onset of pyrene incorporation into the forming micelles, which reduces the I_1_/I_3_ value. At a polymer concentration significantly higher than the CMC, all pyrene transitions into the micelles, and the dependence of the intensity ratio on the polymer concentration reaches a plateau again [84].

Some conclusions can be drawn from the analysis of the second plateau for polymers of different compositions. For the most hydrophilic polymer without C_12_E_10_M units, a relatively small decrease in the I_1_/I_3_ ratio was observed, with the second plateau at about 1.70 units, compared to the initial ratio of 1.84, which is characteristic of pyrene in the aqueous phase (first plateau level). The introduction of 5% C_12_E_10_M units reduces the intensity ratio to 1.54, while for the three most hydrophobic copolymers, the second plateau level is 1.43 and does not depend on the C_12_E_10_M content. Assuming that the second plateau corresponds to the complete transition of pyrene into the micelles, the intensity ratio characterizes the hydrophobicity of the pyrene environment within the micelles [85,86]. From these results, the following assumptions can be made. First, pyrene is molecularly segregated within the micelles, as the formation of multimolecular aggregates in the micelles would not significantly affect the I_1_/I_3_ ratio. The presence of pyrene as individual molecules in the micelles makes it less likely that pyrene acts as a catalyst for aggregation in the studied systems. Second, the investigated polymers tend to form micelle-like aggregates rather than random configurations, where the I_1_/I_3_ ratio would linearly correlate with the polymer hydrophobicity, which is not observed for polymers with a high C_12_E_10_M content. In the case of there being an absence or low content of hydrophobic comonomer units, the micelle core is mainly formed by the relatively hydrophobic backbone compared to the oligoethylene glycol fragment. In this case, the core is small and not highly hydrophobic. With more than 5% C_12_E_10_M content, more hydrophobic cores enriched with alkyl fragments are formed. Further increases in C_12_E_10_M content lead to the formation of cores consisting of alkyl groups. Consequently, the introduction of additional C_12_E_10_M units does not affect the micelle core composition, as reflected in the constant I_1_/I_3_ value at the second plateau.

The CMC values of the polymers were found to be in the range of a few mg/L (Table 4), which is typical for methacrylic polymers with oligoethylene glycol side chains [87,88]. Micelles of the C_1_E_5_M homopolymer form at a polymer concentration of 3 mg/L, while the introduction of cationic hydrophilic DMq units reduces the tendency for aggregation via hydrophobic interactions and increases electrostatic repulsion, resulting in a lower aggregation propensity and higher CMC values. In contrast, the introduction of hydrophobic C_12_E_10_M lowers the CMC due to increased hydrophobic interactions as the driving force for micellization. Along with the decrease in the CMC in the series of terpolymers, the size of the aggregates (hydrodynamic radius *R*_h_) increases from 2.2 to 5.3 nm, indicating an increase in the aggregation number due to enhanced hydrophobic effects. The largest *R*_h_ was observed for the C_1_E_5_M homopolymer, which may be related to its higher molecular weight. The low CMC values make the synthesized polymers promising for use as drug delivery systems.

The monomer composition of the polymers, particularly the number of hydrophobic groups, significantly influences the micelle capacity (Table 4). For the C_1_E_5_M homopolymer and its copolymers with DMq, the capacity ranges from 0.6 to 1.7 mg of pyrene per gram of polymer. In contrast, for terpolymers with alkyl groups, the capacity is significantly higher (from 5.8 to 24.2 mg/g) and increases linearly with the content of C_12_E_10_M units. This is attributed to the formation of more hydrophobic micellar cores and an increase in their volume fraction within the micelles.

Thus, increasing the proportion of the hydrophobic comonomer in the terpolymers reduces interfacial activity at the phase boundary but enhances the polymers’ tendency for micellization and increases the micelles’ capacity for hydrophobic components, which is crucial for drug delivery applications. By varying the proportion of the cationic comonomer, the desired aggregation characteristics of the polymer can be tailored for specific tasks by adjusting hydrophilicity through changes in the hydrophobic comonomer content.

**Figure 4:**
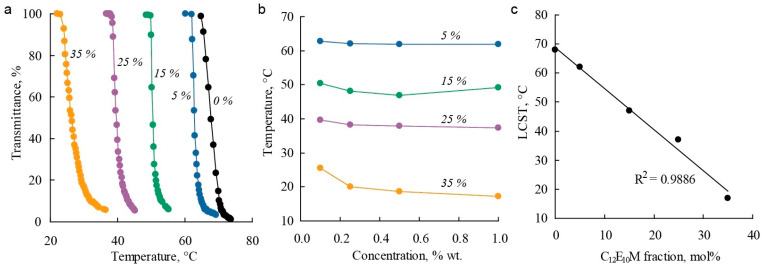
Dependencies of light transmittance on temperature (**a**), critical temperature on polymer concentration (**b**), and LCST values on polymer composition (**c**) for C_1_E_5_M-C_12_E_10_M-DMq polymers with varying contents of C_12_E_10_M units (mol.%) in aqueous solutions.

The influence of the molecular brush composition on thermoresponsive properties in aqueous solutions was studied using turbidimetry. Molecular brushes exhibit thermoresponsive properties with a lower critical solution temperature (LCST). For the C_1_E_5_M homopolymer in distilled water, the LCST was found to be around 64 °C, which is consistent with previously reported data [89]. The introduction of 20% DMq units rendered the copolymer soluble in water across the entire studied temperature range. In aqueous solutions with electrolytes (0.1 M NaCl), critical temperatures were observed, and the copolymer exhibited thermoresponsive properties, indicating the influence of ionic strength on polymer solubility. Figure 4a shows examples of light transmittance curves for polymer solutions with varying C_12_E_10_M content as a function of temperature. All the presented polymers exhibit sharp phase transitions, and the phase transition temperatures show little dependence on concentration within the studied range (Figure 4b). By adjusting the monomer composition, the LCST can be controlled over a wide temperature range: increasing the proportion of DMq raises the critical temperature, while introducing hydrophobic C_12_E_10_M units linearly reduces the LCST (Figure 4c) and narrows the temperature range of polymer solubility.

Thus, it is possible to synthesize terpolymers with varying DMq contents that have an LCST near physiological temperatures (36 °C) by regulating the hydrophilicity of the polymers through changes in the C_12_E_10_M content. This is important for the use of these polymers as micellar drug delivery systems.

**Figure 5:**
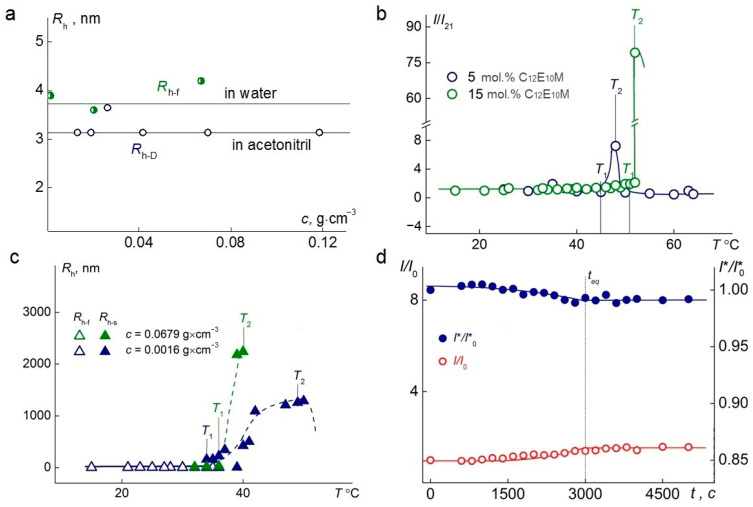
(**a**) The dependence of the hydrodynamic radius on the concentration of terpolymer C_1_E_5_M-C_12_E_10_M-DMq 70:25:5. (**b**) Temperature dependence of the relative light-scattering intensity, *I*/*I*_21_, for aqueous solutions of terpolymers C_1_E_5_M-C_12_E_10_M-DMq with varying contents of C_12_E_10_M units (mol.%) at the concentration c = 0.01 g·cm^−3^. *I*_21_ is the light-scattering intensity at 21 °C. (**c**) Temperature dependences of the hydrodynamic radii *R*_h-f_ and *R*_h-s_ for aqueous solutions of the terpolymer C_1_E_5_M-C_12_E_10_M-DMq 70:25:5 at different concentrations. (**d**) The time-dependent behavior of the *I/I*_0_ for the experiments on the terpolymer C_1_E_5_M-C_12_E_10_M-DMq 70:25:5 at 36 °C, c = 0.002 g·cm^−3^.

A single mode was detected by dynamic light scattering at 21 °C in aqueous solutions at room temperature. For all the samples, within the studied concentration range, the hydrodynamic radii, *R*_h-f_, of the particles in water were, on average, 30% larger than the hydrodynamic radii, *R*_h−D_, of the particles determined in acetonitrile (Figure 5a), which also indicates a multimolecular structure of the particles in water.

When studying C_1_E_5_M-C_12_E_10_M-DMq polymers in aqueous solutions upon heating, the solutions remained transparent up to temperature *T*_1_ (Figure 5b), at which the light-scattering intensity began to increase until temperature *T*_2_. The temperature range from *T*_1_ to *T*_2_ represents the phase separation interval. At *T* < *T*_1_, the intensity, *I*, remains unchanged. Near temperature *T*_1_, aggregates appear in the polymer solutions, and their hydrodynamic radii, *R*_h-s_, increase upon heating in the range from *T*_1_ to *T*_2_ (Figure 5c). At *T* > *T*_2_, both the *I* and *R*_h-s_ decreased with temperature, as the solutions became turbid, and the light scattering was no longer classical (multiple scattering patterns were observed).

The obtained values of the phase separation temperature for aqueous solutions of C_1_E_5_M-C_12_E_10_M-DMq (80:15:5) at concentrations ranging from 0.0025 g∙cm^−3^ to 0.01 g∙cm^−3^ demonstrated that the phase separation temperature decreases slightly with increasing concentration and aligns well with the temperatures determined by turbidimetry. The time, teq, required for the solutions to reach equilibrium values of the relative intensity of scattered light (*I*/*I*_0_) and the relative intensity of optical transmittance (I*/I*_0_) after a temperature change, is relatively short and amounts to 3000 s (Figure 5d).

**Figure 6:**
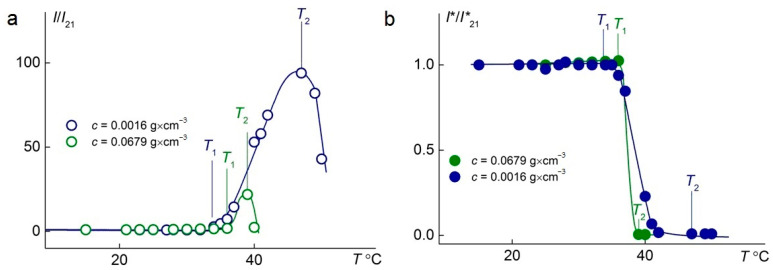
Temperature dependences of relative light-scattering intensity, *I/I*_21_, (**a**) and relative optical transmission, I*/I*_21_, (**b**) for aqueous solutions of C_1_E_5_M-C_12_E_10_M-DMq 70:25:5. *I*_21_ and I*_21_ are light-scattering intensity and optical transmission at 21 °C, respectively.

The temperature-dependent changes in the relative intensities of scattered and transmitted light at different concentrations of the C_1_E_5_M-C_12_E_10_M-DMq (70:25:5) polymer are presented in Figure 6. In both cases, as the concentration increases from 0.11 g/L to 67.9 g/L, a significant reduction in the phase transition width is observed, ensuring a faster response of the polymer to changes in the solution temperature, which is important from a practical standpoint. Additionally, it was found that at lower concentrations, a significantly higher intensity of light scattering is achieved.

Thus, while in acetonitrile, the macromolecules of C_1_E_5_M-C_12_E_10_M-DMq are segregated; in water, the formation of small micelle-like aggregates is observed. However, in both solvents, the polymers are characterized by high intramolecular density. For all the studied polymers in water, phase separation occurs, with the formation of large aggregates of micron size. The polymers exhibit high kinetic performance in thermoresponsive solubility changes in aqueous media, which can be utilized to impart targeted action to micellar drug delivery systems based on these polymers.

## 4. Conclusions

The conducted studies demonstrated the possibility of synthesizing triple polymeric molecular brushes based on hydrophilic, hydrophobic, and cationic monomers using the “grafting-through” method under RAFT polymerization conditions, which ensures polymerization to high conversions while maintaining a high degree of molecular weight control.

The obtained cationic brushes in aqueous solutions exhibited high amphiphilicity, reflected in their significant surface-active properties, tendency to form micellar structures at low concentrations, and thermoresponsive behavior characterized by a lower critical solution temperature. The studied polymer brushes are capable of solubilizing low-molecular-weight hydrophobic substances in aqueous media through their incorporation into the hydrophobic core of micelles. The presented characteristics of the polymer brushes classify them as promising materials for use in controlled drug and gene delivery systems.

A key advantage of the triple polymer brushes is that the number of cationic units in the polymer brush can be tailored depending on the application and specific requirements without compromising other properties. This is achieved by adjusting the proportion of the hydrophobic comonomer, which ensures the preservation of the desired amphiphilicity.

## Figures and Tables

**Table 1:** RAFT polymerization conditions and molecular weight characteristics (SEC) of the polymers.

#	[M_1_]:[M_2_]:[M_3_] ^[a]^	[m_1_]:[m_2_]:[m_3_] ^[b]^	[M]:[CTA]:[I] ^[c]^	X ^[d]^, %	Mth ^[e]^	M_n_	M_w_	M_w_/M_n_
1	100:0:0	100:0:0	100:0:1	90.4	-	27,600	56,200	2.0
2	80:0:20	-	100:0:1	96.5	-	30,500	65,100	2.1
3	95:0:5	94:0:6	100:4:1	88.0	6600	5000	6400	1.3
4	90:5:5	89:5:6	100:4:1	93.8	7900	5000	6200	1.2
5	80:15:5	77:15:8	100:4:1	95.0	8900	4700	6200	1.3
6	70:25:5	68:24:8	100:4:1	85.8	8900	5300	6800	1.3
7	60:35:5	59:35:6	100:4:1	93.0	10,600	8300	10,100	1.2

[a] The initial molar ratio of monomers in the reaction mixture: M_1_—macromonomer C_1_E_5_M; M_2_—macromonomer C_12_E_10_M; M_3_—monomer DMq; [b] the molar ratio of the monomer units in the polymer; [c] the initial molar ratio of the monomers, CTA, and the initiator; [d] the monomer conversion; [e] the theoretical molecular weight calculated using the equation Mth = Mr_monomer_ ∙ X ∙ [M]: [CTA] + Mr_CTA_.

**Table 2:** Solubility of the synthesized polymers.

Solvent	ε	Monomer DMq	Terpolymers	Homopolymer C_1_E_5_M
Hexane	1.9			
Cyclohexane	2.0			
Toluene	2.4			
Chloroform	4.8			
Ethyl acetate	6.0			
Tetrahydrofuran	7.6			
Octanol	10.3			

**Table 3:** Molecular masses (SLS) and hydrodynamic characteristics of the terpolymers in acetonitrile.

[M_1_]:[M_2_]:[M_3_]	*M*_w_, g·mol^−1^ SLS	[η], cm^3^·g^−1^	*R*_h−D_, nm	K_H_	*dn/dc* cm^3^∙g^−1^	*A*_0_ × 10^10^, erg·K^−1^mol^−1/3^	*A*_2_ × 10^−4^, cm^3^·mol·g^−2^
90:5:5	7700	5.4	2.1	0.7	0.14	2.6	3.6
80:15:5	9000	6.3	2.4	0.6	0.14	2.5	3.9
70:25:5	9100	6.6	3.1	0.3	0.14	2.2	4.0
60:35:5	11,000	6.9	4.0	0.3	0.14	2.3	4.2

**Table 4:** Aggregation characteristics of the polymers.

#	[M_1_]:[M_2_]:[M_3_] ^[a]^	LCST, °C	CMC, mg/L	*R*_h_, nm	Loading Capacity, mg/g
1	100:0:0	64	3.0	5.8	0.9
2	80:0:20	>90	9.5	2.0	0.6
3	90:0:5	68	4.3	2.2	1.7
4	90:5:5	62	3.9	2.7	5.8
5	80:15:5	47	1.9	3.1	11.3
6	70:25:5	37	2.8	3.8	20.6
7	60:35:5	17	2.7	5.3	24.2

[a] The molar ratio of the monomers: M_1_—macromonomer C_1_E_5_M; M_2_—macromonomer C_12_E_10_M; M_3_—monomer DMq.

## Data Availability

The data supporting these findings are available in the Supplementary Material.

## Supplementary Materials

The following supporting information can be downloaded at https://www.mdpi.com/article/10.3390/polym17091200/s1: details of the experimental procedures and data from elemental analysis, IR spectroscopy, and NMR spectroscopy.

## References

[1] Imran M., Shah M.R., Shafiullah, Grumezescu A.M. (2018). Amphiphilic block copolymers–based micelles for drug delivery. Design and Development of New Nanocarriers.

[2] Ahmad Z., Shah A., Siddiq M., Kraatz H.-B. (2014). Polymeric micelles as drug delivery vehicles. RSC Adv..

[3] Toscanini M.A., Limeres M.J., Garrido A.V., Cagel M., Bernabeu E., Moretton M.A., Chiappetta D.A., Cuestas M.L. (2021). Polymeric micelles and nanomedicines: Shaping the future of next generation therapeutic strategies for infectious diseases. J. Drug Deliv. Sci. Technol..

[4] Lu Y., Zhang E., Yang J., Cao Z. (2018). Strategies to improve micelle stability for drug delivery. Nano Res..

[5] Wang H., Ding S., Zhang Z., Wang L., You Y. (2019). Cationic micelle: A promising nanocarrier for gene delivery with high transfection efficiency. J. Gene. Med..

[6] Yang L., Song S., Yin M., Yang M., Yan D., Wan X., Xiao J., Jiang Y., Yao Y., Luo J. (2023). Antibiotic-based small molecular micelles combined with photodynamic therapy for bacterial infections. Asian J. Pharm. Sci..

[7] Leng M., Hu S., Lu A., Cai M., Luo X. (2016). The anti-bacterial poly(caprolactone)-poly(quaternary ammonium salt) as drug delivery carriers. Appl. Microbiol. Biotechnol..

[8] Sheiko S.S., Sumerlin B.S., Matyjaszewski K. (2008). Cylindrical molecular brushes: Synthesis, characterization, and properties. Prog. Polym. Sci..

[9] Zhang X., Dai Y. (2019). Recent development of brush polymers via polymerization of poly(ethylene glycol)-based macromonomers. Polym. Chem..

[10] Adeli F., Abbasi F., Ghandforoushan P., Külahlı H.E., Meran M., Abedi F., Ghamkhari A., Afif S. (2023). Recent advances in formulation and application of molecular polymer brushes in biomedicine: Therapeutic, diagnostic, and theranostics capabilities. Nano Today.

[11] Lokesh M.G., Tiwari A.K. (2024). A concise review on polymer brushes and its interaction with surfactants: An approach towards smart materials. J. Mol. Liq..

[12] Müllner M., Müller A.H.E. (2016). Cylindrical polymer brushes—Anisotropic building blocks, unimolecular templates and particulate nanocarriers. Polymer.

[13] Xie G., Martinez M.R., Olszewski M., Sheiko S.S., Matyjaszewski K. (2019). Molecular Bottlebrushes as Novel Materials. Biomacromolecules.

[14] Tu S., Choudhury C.K., Luzinov I., Kuksenok O. (2019). Recent advances towards applications of molecular bottlebrushes and their conjugates. Curr. Opin. Solid State Mater. Sci..

[15] Kang J.-J., Shehu K., Sachse C., Jung F.A., Ko C.-H., Barnsley L.C., Jordan R., Papadakis C.M. (2021). A molecular brush with thermoresponsive poly(2-ethyl-2-oxazoline) side chains: A structural investigation. Colloid. Polym. Sci..

[16] Feng C., Huang X. (2018). Polymer Brushes: Efficient Synthesis and Applications. Acc. Chem. Res..

[17] Lee H.-il, Pietrasik J., Sheiko S.S., Matyjaszewski K. (2010). Stimuli-responsive molecular brushes. Prog. Polym. Sci..

[18] Foster J.C., Varlas S., Couturaud B., Coe Z., O’Reilly R.K. (2019). Getting into Shape: Reflections on a New Generation of Cylindrical Nanostructures’ Self-Assembly Using Polymer Building Blocks. J. Am. Chem. Soc..

[19] Pelras T., Mahon C.S., Müllner M. (2018). Synthesis and Applications of Compartmentalised Molecular Polymer Brushes. Angew. Chem. Int. Ed..

[20] Bai S., Jia D., Ma X., Liang M., Xue P., Kang Y., Xu Z. (2021). Cylindrical polymer brushes-anisotropic unimolecular micelle drug delivery system for enhancing the effectiveness of chemotherapy. Bioact. Mater..

[21] Alsehli M., Gauthier M. (2023). Unimolecular Micelles from Randomly Grafted Arborescent Copolymers with Different Core Branching Densities: Encapsulation of Doxorubicin and In Vitro Release Study. Materials.

[22] Zheng Y., Pan M., Lu C., Liu D. (2018). Polypeptide-based amphiphilic brush copolymers as unimolecular micelles: Synthesis, characterisation, and encapsulation study. Micro Nano Lett..

[23] Müllner M. (2022). Molecular polymer bottlebrushes in nanomedicine: Therapeutic and diagnostic applications. Chem. Commun..

[24] Xie G., Krys P., Tilton R.D., Matyjaszewski K. (2017). Heterografted Molecular Brushes as Stabilizers for Water-in-Oil Emulsions. Macromolecules.

[25] Hsieh T.-L., Martinez M.R., Garoff S., Matyjaszewski K., Tilton R.D. (2021). Interfacial dilatational rheology as a bridge to connect amphiphilic heterografted bottlebrush copolymer architecture to emulsifying efficiency. J. Colloid Interface Sci..

[26] Asadi V., Ruiz-Franco J., van der Gucht J., Kodger T.E. (2023). Tuning moduli of hybrid bottlebrush elastomers by molecular architecture. Mater. Des..

[27] Rao S., Zeng X., Cheng X., Fan J., He D., Ren L., Du G., Zeng X. (2023). Damping, soft, and thermally conductive composite elastomer via introducing bottlebrush chains. Chem. Eng. J..

[28] Gao Q., Yu M., Su Y., Xie M., Zhao X., Li P., Ma P.X. (2017). Rationally designed dual functional block copolymers for bottlebrush-like coatings: In vitro and in vivo antimicrobial, antibiofilm, and antifouling properties. Acta Biomater..

[29] Stevens M.C., Taylor N.M., Guo X., Hussain H., Mahmoudi N., Cattoz B.N., Leung A.H.M., Dowding P.J., Vincent B., Briscoe W.H. (2024). Diblock bottlebrush polymer in a non-polar medium: Self-assembly, surface forces, and superlubricity. J. Colloid Interface Sci..

[30] Zhuo C., You H., Gao F., Liu S., Wang X., Wang F. (2023). Bottlebrush polymeric catalyst: Boosting activity for CO_2_/epoxide copolymerization. Fuel.

[31] Huang J., Zhu X., Wang Y., Min Y., Li X., Zhang R., Qi D., Hua Z., Chen T. (2022). Compartmentalization of incompatible catalysts by micelles from bottlebrush copolymers for one-pot cascade catalysis. Polymer.

[32] Li C., Wang J., Wang Y., Gao H., Wei G., Huang Y., Yu H., Gan Y., Wang Y., Mei L. (2019). Recent progress in drug delivery. Acta Pharm. Sin. B..

[33] Stolnik S., Illum L., Davis S.S. (1995). Long circulating microparticulate drug carriers. Adv. Drug Del. Rev..

[34] Elezaby R.S., Gad H.A., Metwally A.A., Geneidi A.S., Awad G.A. (2017). Self-assembled amphiphilic core-shell nanocarriers in line with the modern strategies for brain delivery. J. Controlled Release.

[35] Lutz J.-F., Akdemir Ö., Hoth A. (2006). Point by Point Comparison of Two Thermosensitive Polymers Exhibiting a Similar LCST:  Is the Age of Poly(NIPAM) Over?. J. Am. Chem. Soc..

[36] Kazantsev O.A., Orekhov D.V., Simagin A.S., Kamorin D.M., Sivokhin A.P., Savinova M.V., Arifullin I.R., Kavtrova V.D., Lobayev A.N. (2024). Oligo(ethylene glycol) methacrylate-based molecular bottlebrushes: Correlations between composition and phase transition temperatures in aqueous solutions. Eur. Polym. J..

[37] Sivokhin A., Orekhov D., Kazantsev O., Sivokhina O., Orekhov S., Kamorin D., Otopkova K., Smirnov M., Karpov R. (2022). Random and Diblock Thermoresponsive Oligo(ethylene glycol)-Based Copolymers Synthesized via Photo-Induced RAFT Polymerization. Polymers.

[38] Terashima T., Sugita T., Fukae K., Sawamoto M. (2014). Synthesis and Single-Chain Folding of Amphiphilic Random Copolymers in Water. Macromolecules.

[39] Terashima T., Sugita T., Sawamoto M. (2015). Single-chain crosslinked star polymers via intramolecular crosslinking of self-folding amphiphilic copolymers in water. Polym. J..

[40] Hirai Y., Terashima T., Takenaka M., Sawamoto M. (2016). Precision Self-Assembly of Amphiphilic Random Copolymers into Uniform and Self-Sorting Nanocompartments in Water. Macromolecules.

[41] Matsumoto M., Takenaka M., Sawamoto M., Terashima T. (2019). Self-assembly of amphiphilic block pendant polymers as microphase separation materials and folded flower micelles. Polym. Chem..

[42] Kimura Y., Terashima T., Sawamoto M. (2017). Self-Assembly of Amphiphilic Random Copolyacrylamides into Uniform and Necklace Micelles in Water. Macromol. Chem. Phys..

[43] Hattori G., Hirai Y., Sawamoto M., Terashima T. (2017). Self-assembly of PEG/dodecyl-graft amphiphilic copolymers in water: Consequences of the monomer sequence and chain flexibility on uniform micelles. Polym. Chem..

[44] Kowollik B. (2008). Handbook of RAFT Polymerization.

[45] Matyjaszewski K., Sumerlin B.S. (2012). Progress in Controlled Radical Polymerization: Materials and Applications.

[46] Pan Y., Wang X., Yin Z. (2019). Synthesis and evaluation of cationic polymeric micelles as carriers of lumbrokinase for targeted thrombolysis. Asian J. Pharm. Sci..

[47] Dalal R.J., Kumar R., Ohnsorg M., Brown M., Reineke T.M. (2021). Cationic Bottlebrush Polymers Outperform Linear Polycation Analogues for pDNA Delivery and Gene Expression. ACS Macro Lett..

[48] Dey D., Maiti C., Maiti S., Dhara D. (2015). Interaction between calf thymus DNA and cationic bottle-brush copolymers: Equilibrium and stopped-flow kinetic studies. Phys. Chem. Chem. Phys..

[49] Modra K., Dai S., Zhang H., Shi B., Bi J. (2015). Polycation-mediated gene delivery: Challenges and considerations for the process of plasmid DNA transfection. Eng. Life Sci..

[50] Skandalis A., Selianitis D., Pispas S. (2021). PnBA-b-PNIPAM-b-PDMAEA Thermo-Responsive Triblock Terpolymers and Their Quaternized Analogs as Gene and Drug Delivery Vectors. Polymers.

[51] Zheng W., Anzaldua M., Arora A., Jiang Y., McIntyre K., Doerfert M., Winter T., Mishra A., Ma H., Liang H. (2020). Environmentally Benign Nanoantibiotics with a Built-in Deactivation Switch Responsive to Natural Habitats. Biomacromolecules.

[52] Senkum H., Gramlich W.M. (2020). Cationic Bottlebrush Polymers from Quaternary Ammonium Macromonomers by Grafting-Through Ring-Opening Metathesis Polymerization. Macromol. Chem. Phys..

[53] Jiao Y., Niu L.-n., Ma S., Li J., Tay F.R., Chen J.-h. (2017). Quaternary ammonium-based biomedical materials: State-of-the-art, toxicological aspects and antimicrobial resistance. Prog. Polym. Sci..

[54] Kazantsev O.A., Kamorin D.M., Sivokhin A.P., Samodurova S.I., Orekhov D.V., Korotkova T.V. (2014). Copolymerization of amine-containing monomers and dodecyl (meth)acrylate in toluene: Controlling compositional heterogeneity. J. Polym. Res..

[55] Shahrbabaki Z., Oveissi F., Farajikhah S., Ghasemian M.B., Jansen-van Vuuren R.D., Jessop P.G., Yun J., Dehghani F., Naficy S. (2022). Electrical Response of Poly(N-[3-(dimethylamino)propyl] Methacrylamide) to CO_2_ at a Long Exposure Period. ACS Omega.

[56] Kamorin D.M., Kazantsev O.A., Simagin A.S., Orekhov D.V., Savinova M.V., Arifullin I.R., Sivokhin A.P. (2024). Effect of Composition of Nonionic and Cationic Copolymers of Alkoxyoligo(ethylene glycol) Methacrylates and Dodecyl Methacrylate on Their Properties in Solutions. Polym. Sci. Ser. A.

[57] Duan J., Huang Y., Zong S., Jiang J. (2021). Preparation and Drug Release Properties of a Thermo Sensitive GA Hydrogel. Polymers.

[58] Mishra R.K., Ray A.R. (2011). Synthesis and characterization of poly{N-[3-(dimethylamino) propyl] methacrylamide-co-itaconic acid} hydrogels for drug delivery. J. Appl. Polym. Sci..

[59] Moad G., Chong Y.K., Postma A., Rizzardo E., Thang S.H. (2005). Advances in RAFT polymerization: The synthesis of polymers with defined end-groups. Polymer.

[60] Wesslén B., Wesslén K.B. (1989). Preparation and properties of some water-soluble, comb-shaped, amphiphilic polymers. J. Polym. Sci. Part A Polym. Chem..

[61] Wilkinson M.C. (1972). Extended use of, and comments on, the drop-weight (drop-volume) technique for the determination of surface and interfacial tensions. J. Colloid Interface Sci..

[62] Kuckling D., Doering A., Krahl F., Arndt K.F., Matyjaszewski K., Möller M. (2012). Stimuli-Responsive Polymer Systems. Polymer Science: A Comprehensive Reference.

[63] Zhang Q., Weber C., Schubert U.S., Hoogenboom R. (2017). Thermoresponsive polymers with lower critical solution temperature: From fundamental aspects and measuring techniques to recommended turbidimetry conditions. Mater. Horiz..

[64] Zhao C.L., Winnik M.A., Riess G., Croucher M.D. (1990). Fluorescence probe techniques used to study micelle formation in water-soluble block copolymers. Langmuir.

[65] Zengin A., Yildirim E., Caykara T. (2013). RAFT-mediated synthesis and temperature-induced responsive properties of poly(2-(2-methoxyethoxy)ethyl methacrylate) brushes. J. Polym. Sci. Part A Polym. Chem..

[66] Krivorotova T., Grigelis R., Švėgždienė J., Makuška R. (2011). Synthesis of anionic amphiphilic molecular brushes by conventional free-radical and RAFT terpolymerizations. Chemija.

[67] Shi Y., van den Dungen E.T.A., Klumperman B., van Nostrum C.F., Hennink W.E. (2013). Reversible Addition–Fragmentation Chain Transfer Synthesis of a Micelle-Forming, Structure Reversible Thermosensitive Diblock Copolymer Based on the N-(2-Hydroxy propyl) Methacrylamide Backbone. ACS Macro Lett..

[68] Yuan Y., Luo Z., Chen J., He C., Hao K., Tian H. (2024). Constructing thermoresponsive PNIPAM-based microcarriers for cell culture and enzyme-free cell harvesting. Chin. Chem. Lett..

[69] Tsvetkov V.N. (1989). Rigid-Chain Polymers: Hydrodynamic and Optical Properties in Solution.

[70] Tsvetkov V.N., Lavrenko P.N., Bushin S.V. (1984). Hydrodynamic invariant of polymer molecules. J. Polym. Sci. Polym. Chem. Ed..

[71] Tsvetkov V.N., Lavrenko P.N., Bushin S.V.e. (1982). A hydrodynamic invariant of polymeric molecules. Russ. Chem. Rev..

[72] Burchard W., Roovers J. (1999). Solution Properties of Branched Macromolecules. Branched Polymers II.

[73] Simonova M., Simagin A., Kamorin D., Orekhov S., Filippov A., Kazantsev O. (2022). The Solution Properties of Polymethacrylate Molecular Brushes with Oligo(ethylene glycol) and Oligo(propylene glycol) Side Chains. Polymers.

[74] Simonova M., Ilgach D., Kaskevich K., Nepomnyashaya M., Litvinova L., Filippov A., Yakimansky A. (2021). Novel Amphiphilic Polyfluorene-Graft-(Polymethacrylic Acid) Brushes: Synthesis, Conformation, and Self-Assembly. Polymers.

[75] Simonova M., Kamorin D., Sadikov A., Filippov A., Kazantsev O. (2022). The Influence of Synthesis Method on Characteristics of Buffer and Organic Solutions of Thermo- and pH-Responsive Poly(N-[3-(diethylamino)propyl]methacrylamide)s. Polymers.

[76] Simonova M.A., Tarasova E.V., Dudkina M.M., Tenkovtsev A.V., Filippov A.P. (2019). Synthesis and hydrodynamic and conformation properties of star-shaped polystyrene with calix[8]arene core. Int. J. Polym. Anal. Charact..

[77] Schreier S., Malheiros S.V.P., de Paula E. (2000). Surface active drugs: Self-association and interaction with membranes and surfactants. Physicochemical and biological aspects. BBA Biomembr..

[78] Sun B., Wang P., Shao C., Jiang P., Guo Y., Yan S., Fang W. (2025). Amphiphilic comb-block copolymers synthesized by photoinitiated polymerization for stabilization of oil–water emulsion by solution self-assembly. Fuel.

[79] Yang H., Lv Z., Zhang M., Jiang J., Xu B., Shen J., Jiang H., Kang W. (2023). A novel active amphiphilic polymer for enhancing heavy oil recovery: Synthesis, characterization and mechanism. J. Mol. Liq..

[80] Kazantsev O.A., Kamorin D.M., Orekhov D.V., Sivokhin A.P. (2015). Study of amphiphilic properties of amine- and oligo(ethylene glycol)-containing (meth)acrylic monomers. Des. Monomers Polym..

[81] Zhou M., Bi Y., Zhou H., Chen X., Zhang F., Li Y., Qu X. (2021). Aggregation Behavior of Poly(Acrylic acid-co-Octadecyl Methacrylate) and Bovine Serum Albumin in Aqueous Solutions. ChemistryOpen.

[82] Hong Z., Yongjun M., Hang W., Lin X. (2010). Rheological Properties of Hydrophobically Modified Poly(acrylic acid) in Mixed Solutions. J. Solution Chem..

[83] Banjare R.K., Banjare M.K., Behera K., Tandon M., Pandey S., Ghosh K.K. (2021). Deep eutectic solvents as modulator on the micellization behaviour of cationic surfactants and potential application in human serum albumin aggregation. J. Mol. Liq..

[84] Gyulai G., Magyar A., Rohonczy J., Orosz J., Yamasaki M., Bosze S.z., Kiss É. (2016). Preparation and characterization of cationic Pluronic for surface modification and functionalization of polymeric drug delivery nanoparticles. Express Polym. Letters.

[85] Urbano B., Silva P., Olea A.F., Fuentes I., Martinez F. (2008). Self-assembly of triblock copolymers in aqueous solution. J. Chil. Chem. Soc..

[86] Wang S., Liu C., Chen H., Zhu A.D., Qian F. (2020). Impact of Surfactants on Polymer Maintained Nifedipine Supersaturation in Aqueous Solution. Pharm. Res..

[87] Hibino M., Tanaka K., Ouchi M., Terashima T. (2022). Amphiphilic Random-Block Copolymer Micelles in Water: Precise and Dynamic Self-Assembly Controlled by Random Copolymer Association. Macromolecules.

[88] Topuzogullari M., Bulmus V., Dalgakiran E., Dincer S. (2014). pH- and temperature-responsive amphiphilic diblock copolymers of 4-vinylpyridine and oligoethyleneglycol methacrylate synthesized by RAFT polymerization. Polymer.

[89] Lutz J.-F. (2008). Polymerization of oligo(ethylene glycol) (meth)acrylates: Toward new generations of smart biocompatible materials. J. Polym. Sci. Part A Polym. Chem..

