# Supplementary material for: Ammonium-Containing Methacrylic Polymer Brushes with Adjustable Hydrophilicity: Synthesis and Properties in Aqueous Solutions
Source: Polymers (Basel). 2025 Apr 27;17(9):1200. doi: 10.3390/polym17091200 (PMC12073613; doi:10.3390/polym17091200)
Supplement: Supplementary file 1 [file polymers-17-01200-s001.zip › polymers-3612774-supplementary.pdf]

Ammonium-Containing Methacrylic Polymer Brushes with Adjustable Hydrophilicity: Synthesis and Properties in Aqueous Solutions

Denis Kamorin 1,*, Alexander Simagin 1, Oleg Kazantsev 1, Maria Savinova 1, Maria Simonova 2, Denis Sadkov 2, Ildar Arifullin 1 and Yaroslav Dolinov 1

1 Research Laboratory "New Polymeric Materials", Nizhny Novgorod State Technical University n.a. R.E. Alekseev, 24 Minin Street, 603950 Nizhny Novgorod, Russia

2 Laboratory of Biomimetic Polymer Materials, Branch of Petersburg Nuclear Physics Institute Named by B.P. Konstantinov of National Research Centre «Kurchatov Institute»—Institute of Macromolecular Compounds, Bolshoy Prospekt 31, 199004 Saint Petersburg, Russia

* Correspondence: d.kamorin@mail.ru

Experimental section

The cationic methacrylic monomer N-methacryloylaminopropyl-N,N-dimethyl-N-propylammonium bromide (DMq) was synthesized in a stirred reactor by alkylating N-[3-(dimethylamino)propyl]methacrylamide (DMAPMA, 99.0 wt.%, Sigma Aldrich) with propyl bromide (reagent grade) in dimethylformamide (reagent grade) at 60 °C for 5 hours. The conversion of DMAPMA, determined by gas chromatography, was 85.2%. The product was precipitated in ethyl acetate. The structure of DMq was confirmed by ^{1}H NMR spectroscopy. Characteristic signals of the ^{1}H NMR spectrum of DMq ($CDCl_3$), δ ppm: t. 7.86, J = 8 (1 H, =NH), m. 5.28-5.31 (2 H, =CH2 vinyl group), m. 3.61-3.69 (2 H, -NH-CH2-CH2-), m. 3.30-3.45 (4 H, -(CH2)2-N-(CH3)2), s. 3.23 (6 H, -(CH2)2-N-(CH3)2), m. 2.05-2.15 (2 H, -NH-CH2-CH2-), m. 1.90-1.95 (3 H, CH2=C-CH3), m. 1.68-1.80 (2 H, -CH2-CH3), t. 0.96, J = 8 (3 H, -CH2-CH3).

Dodecyl methacrylate was obtained by esterification of methacrylic acid with dodecanol (1:1.02 molar ratio) in a round-bottom flask equipped with a reflux condenser and a Dean-Stark trap (to remove water as an azeotrope with the solvent) at 120-125 °C in toluene (40 wt.% solvent) in the presence of p-toluenesulfonic acid (2.0 wt.%) as a catalyst and a radical polymerization inhibitor (hydroquinone, 0.1 wt.%). After removing toluene, the product was isolated by vacuum distillation (boiling point = 120 °C at 400 Pa).

The RAFT agent 4-cyano-4-(dodecylsulfanylthiocarbonyl)sulfanylpentanoic acid was synthesized according to the method described in [Polymer, 2005, 46, 8458]. In the first step, bis(dodecylsulfanylthiocarbonyl)disulfide was obtained by reacting potassium tert-butoxide, lauryl mercaptan, iodine, and carbon disulfide in a mixture of organic solvents. In the second step, the target compound was obtained by reacting the intermediate with 4,4'-azobis(4-cyanopentanoic acid). The

product was recrystallized from heptane, washed with distilled water, and dried under vacuum. According to liquid chromatography the purity of the RAFT agent was 96.5 wt.%.

During polymerization, the current concentrations of monomers were determined by chromatography, and conversions were calculated based on their consumption. The content of methacrylic esters in the reaction mixtures was determined by gas chromatography using a "Chromos GC-1000" instrument (Russia, Dzerzhinsk) with a "VB-1" capillary column (0.32 mm × 30 m).

The molecular weight characteristics of the polymers were determined by size-exclusion chromatography using a "Chromos LC-301" instrument (Russia, Dzerzhinsk) with a Waters 410 refractive index detector. The molecular weights of the C_1E_5M homopolymer and the C_1E_5M-DMq copolymer were determined using a PolySep Linear column (Phenomenex, USA) with water as the mobile phase and polyethylene glycol standards. The molecular weight characteristics of the terpolymers were obtained using two Phenogel 10^4 Å and 10^3 Å columns (Phenomenex, USA). Polystyrene standards were used, with tetrahydrofuran as the eluent.

An image of the polymer synthesis reactor is shown in Figure S1.

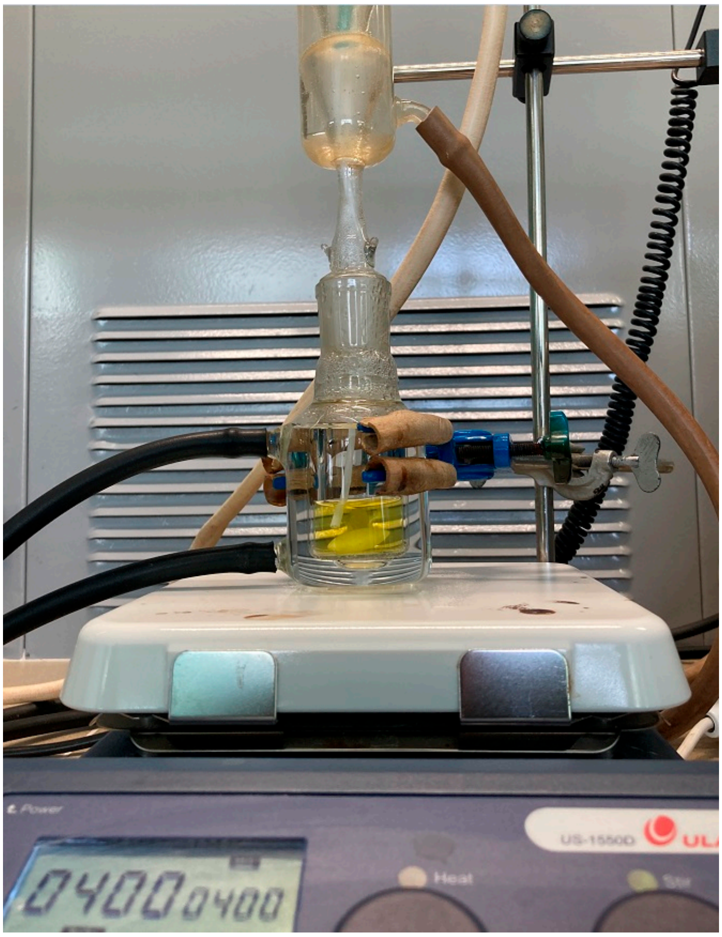

Figure S1. Polymerization reactor.

Supporting Data

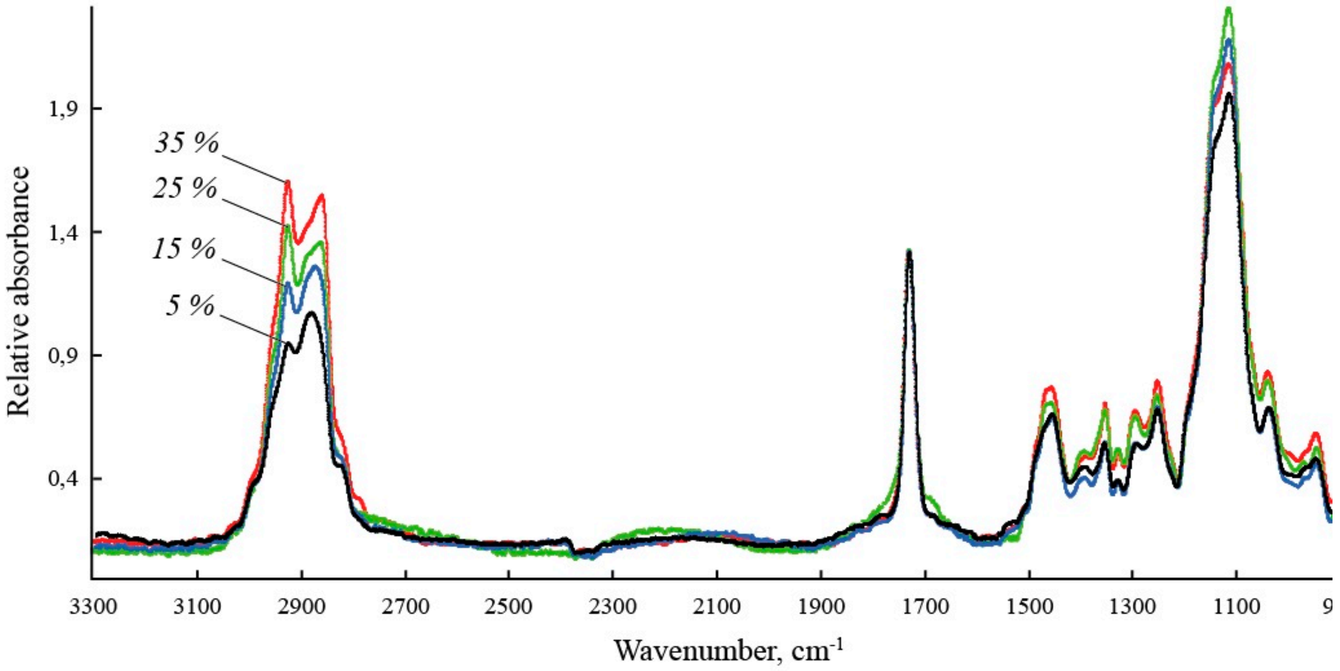

Figure S2. Normalized IR spectra of copolymers with different $C_{12}E_{10}M$ units content, % mol.

Table S1. Elemental composition of the polymers

$[M_1]:[M_2]:[M_3]$ [a]	C, %	H, %	N, %	S, %
90:5:5	55.4 ± 5.5	9.77 ± 0.98	0.63 ± 0.06	0.41 ± 0.04
80:15:5	57.5 ± 5.8	9.48 ± 0.95	0.70 ± 0.07	0.43 ± 0.04
70:25:5	58.2 ± 5.8	10.5 ± 1.1	0.63 ± 0.06	0.40 ± 0.04
60:35:5	58.1 ± 5.8	10.6 ± 1.1	0.44 ± 0.04	0.40 ± 0.04

[a] Molar ratio of monomers: M_1 - macromonomer C_1E_5M; M_2 - macromonomer $C_{12}E_{10}M$; M_3 – monomer DMq.

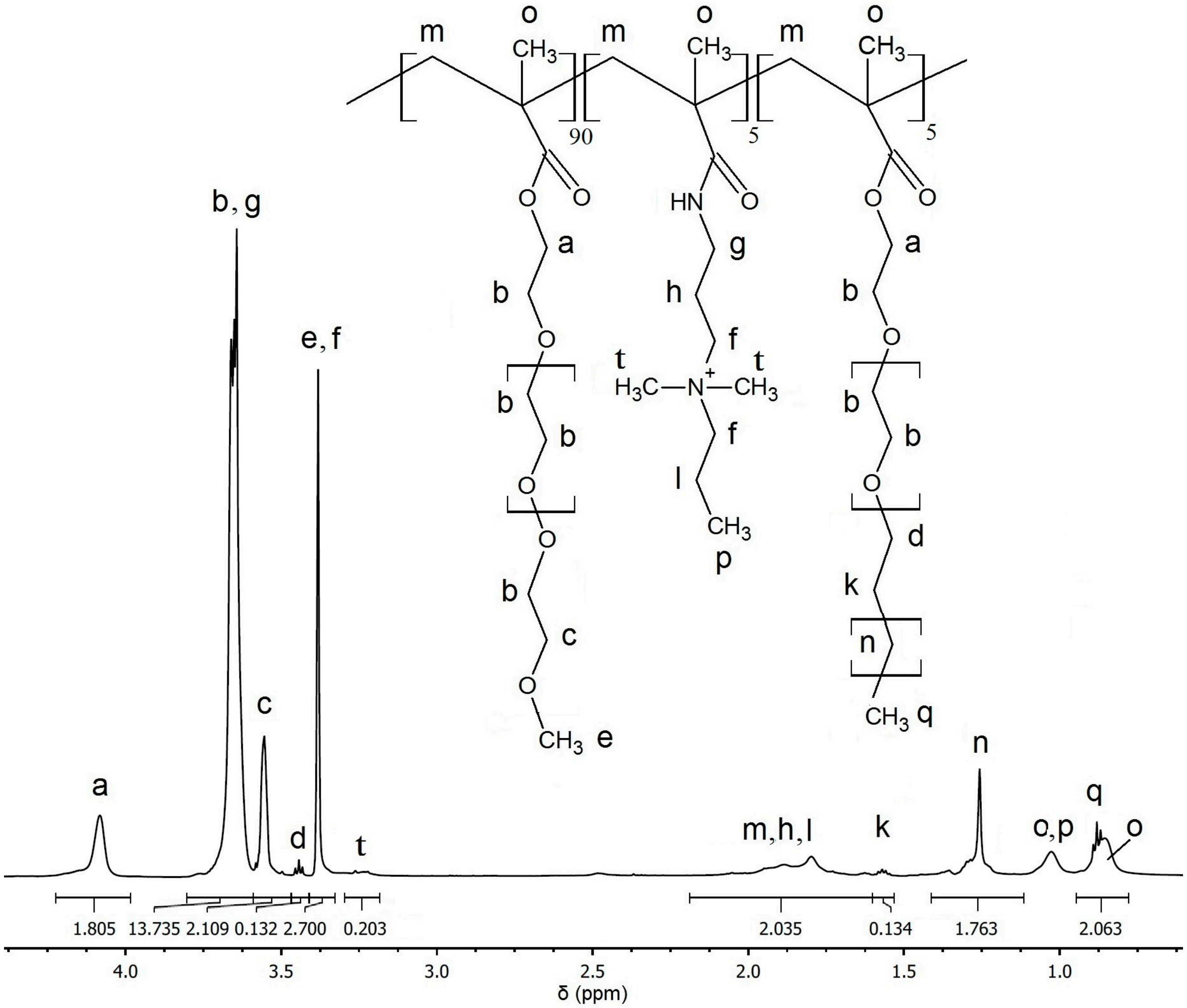

Figure S3. The ^{1}H NMR spectrum of the copolymer with 5 % mol $C_{12}E_{10}M$ units content.

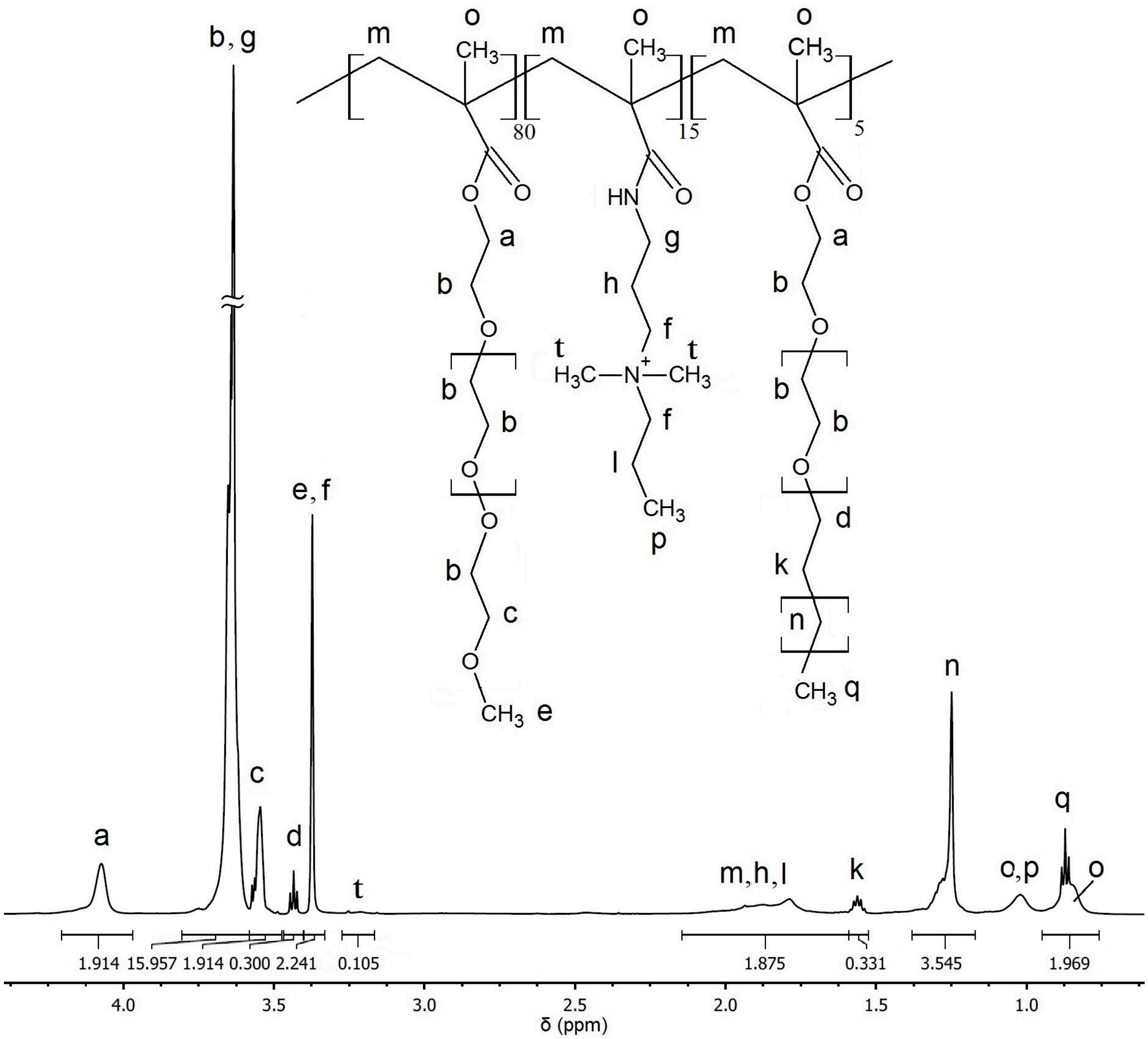

Figure S4. The ^{1}H NMR spectrum of the copolymer with 15 % mol $C_{12}E_{10}M$ units content.

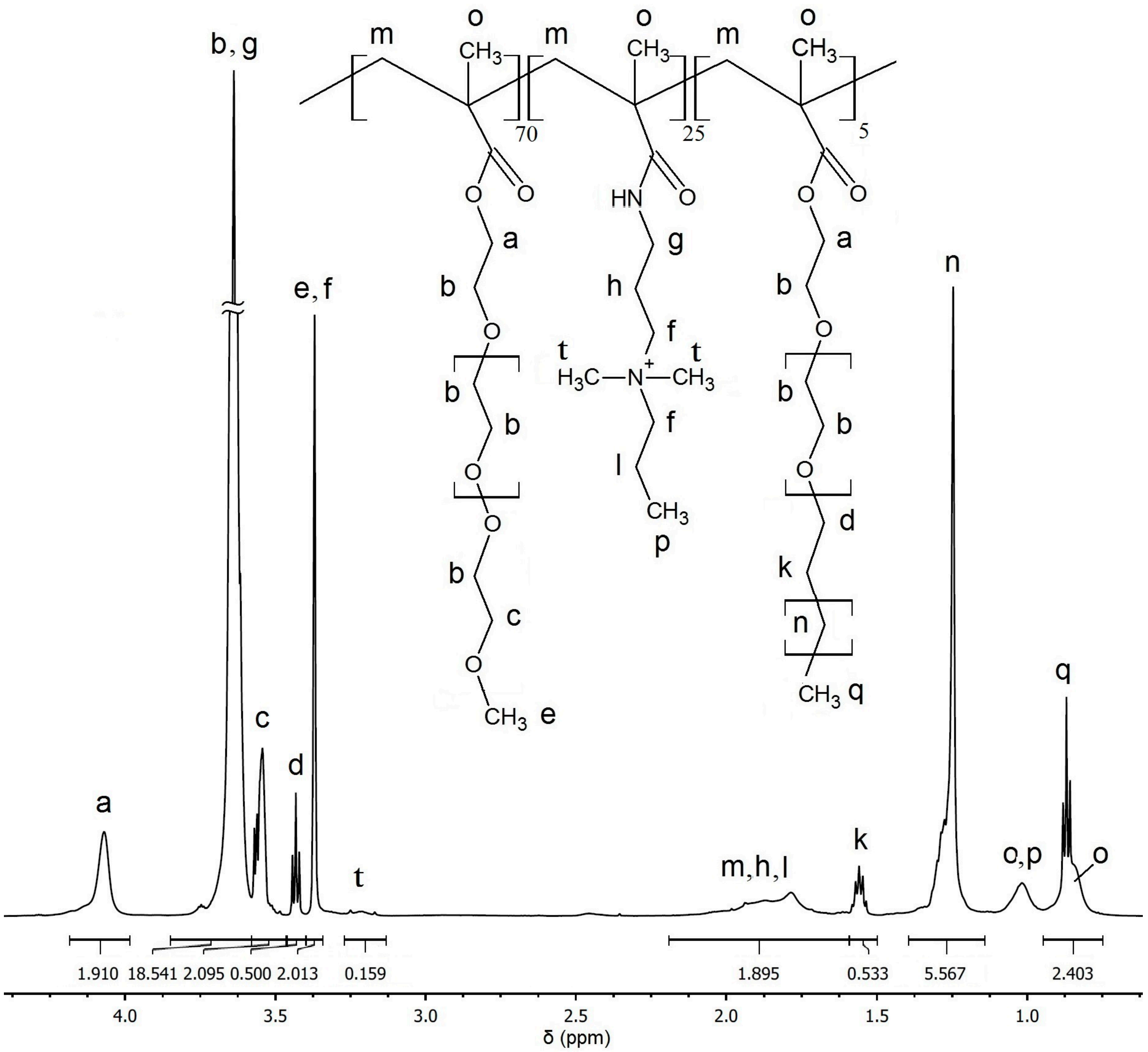

Figure S5. The ^{1}H NMR spectrum of the copolymer with 25 % mol $C_{12}E_{10}M$ units content.

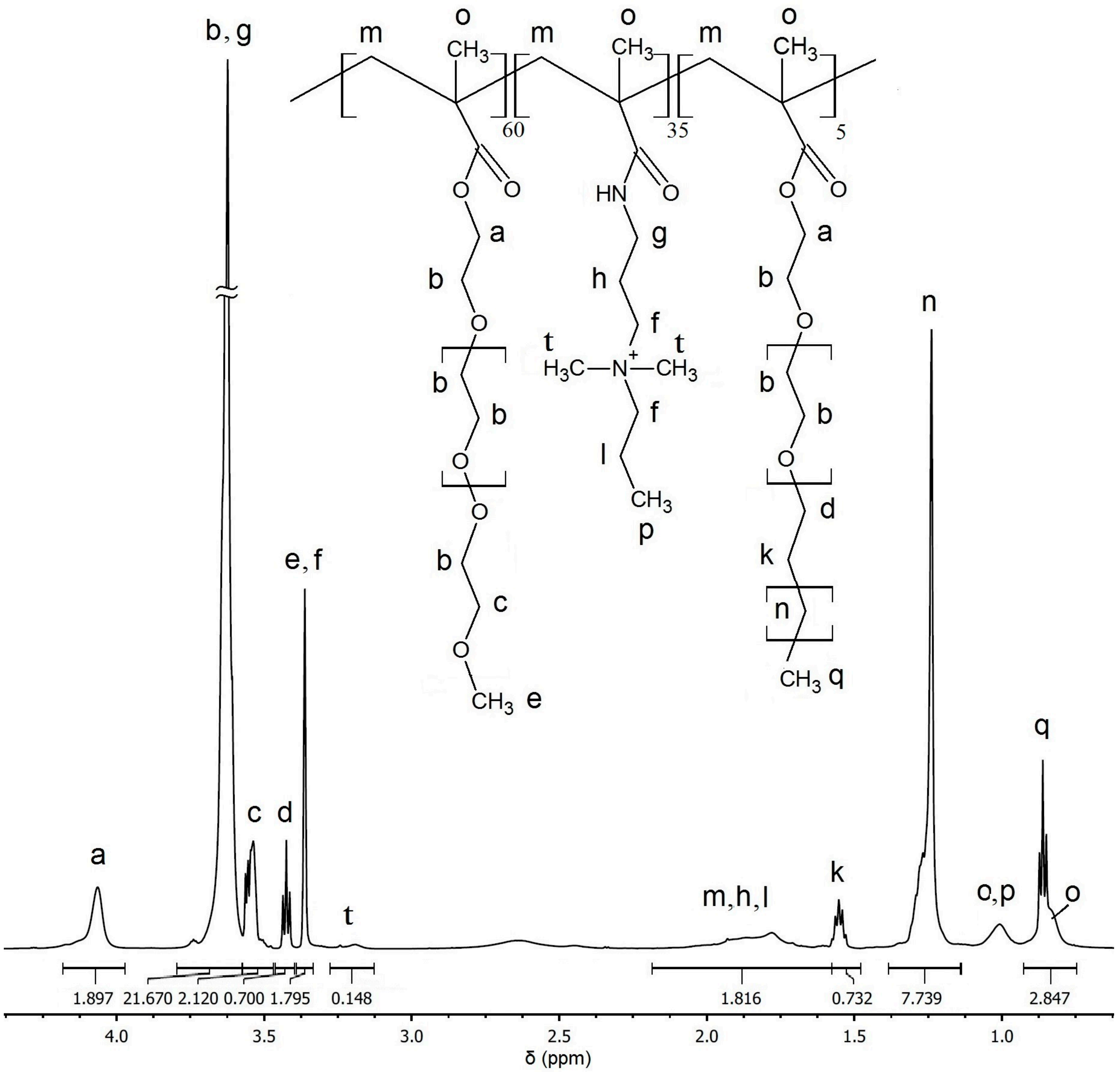

Figure S6. The ^{1}H NMR spectrum of the copolymer with 35 % mol $C_{12}E_{10}M$ units content.
